# The effectiveness of educational interventions in reducing negative attitudes and stigmatisation toward patients with anorexia nervosa

**DOI:** 10.1186/2050-2974-1-S1-O38

**Published:** 2013-11-14

**Authors:** Amy Bannatyne, Peta Stapleton

**Affiliations:** 1 Bond University, Australia

## 

It is frequently reported that clinicians across a range of professional disciplines experience strong negative reactions toward patients with eating disorders, particularly anorexia nervosa (AN). As research consistently demonstrates fear of stigma is the most frequently cited reason explaining why individuals with mental illness do not seek treatment, the current study aimed to develop, evaluate and compare the effectiveness of two differing educational interventions, based on an etiological framing model, against a wait-list control. Participants were fourth-year medicine students randomly assigned to one of three conditions. A three-hour educational workshop was delivered to participants at the beginning of an eight-week clinical rotation. Outcome attitudinal data were collected pre-intervention, post-intervention, and at an eight-week follow-up period. It was hypothesised that both intervention groups would result in more positive attitudes toward AN, compared to the wait-list control, with the biologically-framed intervention resulting in the greatest stigma-reduction effect, consistent with Attribution Theory. Preliminary findings will be discussed.

This abstract was presented in the **Prevention** stream of the 2013 ANZAED Conference.

